# Identification of genetic variants in B cell expressed genes associated with systemic lupus erythematosus in African American females

**DOI:** 10.1093/immhor/vlag029

**Published:** 2026-07-21

**Authors:** Rachel Kidwell, Mansi Gupta, Jake Fontaine, Ignacio Sanz, Christopher D Scharer

**Affiliations:** Department of Microbiology and Immunology, School of Medicine, Emory University, Atlanta, GA, United States; Division of Rheumatology, Department of Medicine, School of Medicine, Emory University, Atlanta, GA, United States; Department of Microbiology and Immunology, School of Medicine, Emory University, Atlanta, GA, United States; Division of Rheumatology, Department of Medicine, School of Medicine, Emory University, Atlanta, GA, United States; Department of Biology, Emory University, Atlanta, GA, United States; Division of Rheumatology, Department of Medicine, School of Medicine, Emory University, Atlanta, GA, United States; Lowance Center for Human Immunology, School of Medicine, Emory University, Atlanta, GA, United States; Department of Microbiology and Immunology, School of Medicine, Emory University, Atlanta, GA, United States

**Keywords:** A20, B cells, polymorphisms, SLE

## Abstract

Systemic lupus erythematosus (SLE) is a complex autoimmune disease characterized by dysregulated B cell responses and pathogenic autoantibody production. Given the central role that B cells play in SLE pathogenesis, we aimed to characterize genetic variation within the B cell compartment of African ancestry (AFR) women with SLE who are disproportionately and more severely affected by SLE. Here, we analyzed RNA-seq data from a previously published study of high disease activity AFR SLE individuals and healthy controls (HC) to identify coding variants in B cells with potential functional consequence that may underpin disease. In this cohort, we identified 575 missense variants associated with 158 unique genes. While many variant-containing genes did not exhibit large transcriptional changes between cohorts, a subset occurred within genes involved in B cell signaling, activation, and differentiation. However, genes enriched in AFR SLE were consistently upregulated across multiple B cell subtypes, suggesting that variant-associated transcriptional changes occur broadly across the B cell compartment and not within a single subset. Among these, the *TNFAIP3* variant rs2230926 was enriched in AFR SLE individuals and occurs at a substantially higher allele frequency in AFR populations. This study provides insights into genetic variation found within the B cell compartment that may contribute to immune dysregulation and disease pathogenesis in SLE in AFR populations.

## Graphical Abstract

**Graphical Abstract:**
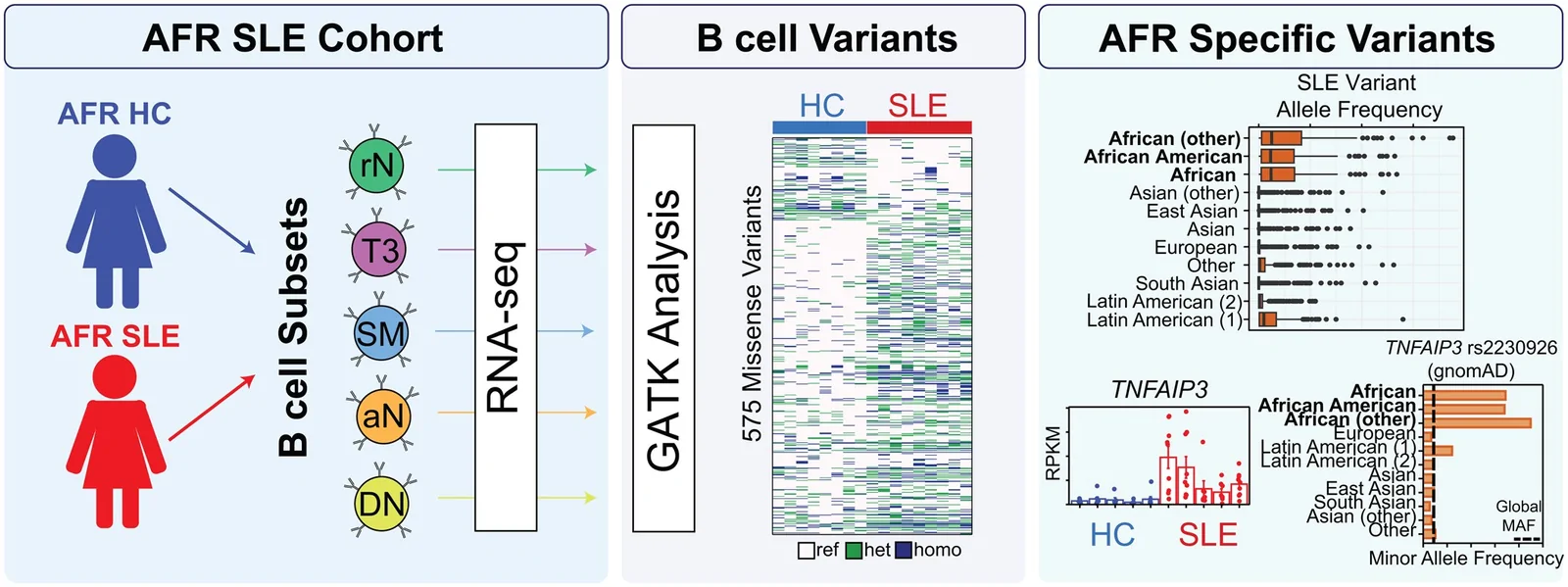

## Introduction

Systemic lupus erythematosus (SLE) is a systemic autoimmune disease characterized by widespread inflammation, the loss of immunologic tolerance to nuclear self-antigens, production of pathogenic autoantibodies, and an impaired clearance of immune complexes, which can be fatal.^1–3^ SLE is a heterogenous disease with clinical variations between individuals; however, it is underpinned by a pronounced sex-bias, with women being approximately 9 times more likely to develop the disease than men.^4^^,^^5^ Despite an overall female dominance, disease incidence is unevenly distributed between women from different ancestral populations with the annual rate of SLE in Black females in the United States much higher compared to White females (reported as high as 15.9 vs 5.7 per 100,000 person-years).^6–8^

The etiology of SLE is complex, with a combination of environmental triggers, immune dysregulation, and genetic susceptibility is believed to contribute to disease.^9–11^ Genome-wide association studies (GWAS) have identified over 100 genetic loci linked to SLE susceptibility,^12–16^ with many disease-associated variants located in non-coding regions of the genome with undefined functional consequences.^17^^,^^18^ However, coding variants have the potential to directly alter protein function and may contribute to immune dysregulation in SLE and be more phenotypically penetrant compared to non-coding. Gain-of-function mutations in Toll-like receptor (TLR) 7 appear to result in SLE and demonstrate the role of TLR signaling in disease.^19^ Furthermore, genetic studies have historically focused on populations of European ancestry with a majority of identified genetic risk loci derived from studies of these cohorts, with minimal associations reported in African ancestry populations (AFR).^20–23^ This imbalance has hindered the discovery of ancestry-associated risk variants for populations with the highest likelihood of developing severe disease.

While genetic susceptibility contributes to SLE risk, the disease manifests clinically across multiple organs and its pathogenesis reflects a failure of immune tolerance mechanisms that normally minimize aberrant lymphocyte activation, particularly within the B cell compartment.^11^^,^^24–26^ The B cell compartment is composed of a diverse population of CD19^+^ cells, which span varying developmental and activation states that give rise to memory B cells and antibody secreting plasma cells (PC). When tolerance checkpoints, such as negative selection, fail, autoreactive B cells survive while chronic engagement of nucleic acid-sensing pathways, type I interferon signaling (IFN), and NF-κB activation promote their activation and differentiation.^27–29^ Beyond autoantibody secretion, dysregulation in the composition of the B cell compartment results in expanded extrafollicular (EF) subsets associated with rapid differentiation of autoreactive B cells. Most prominently is the expansion of IgD^–^CD27^–^ double negative (DN2) B cells that also lack CXCR5 and express CD11c.^9^^,^^28^^,^^30^^,^^31^ In addition, DN2 B cells have been associated with an increased sensitivity to TLR signaling and an increased production of proinflammatory cytokines, which may influence the disease state in SLE.^28^ Together, these findings place B cells and PC at the center of SLE pathogenesis and suggest that genetic variants impacting B cell activation, differentiation, and tolerance may underpin disease risk.

In this study, we sought to identify SLE genetic risk variants in a high disease activity cohort of Black AFR females undergoing disease flares. Analyzing RNA-seq data of defined B cell subsets, including resting naive (rN), transitional (T3), activated naive (aN), switched memory (SM), and double negative (DN2), we identified 575 missense variants associated with 158 genes within the B cell compartment and AFR SLE. Furthermore, variant discovery partnered with transcriptional analysis revealed that only a small subset of variant-containing genes was differentially expressed and several variants occurred within genes involved in TLR and immune signaling pathways relevant to B cell activation and differentiation. Together, these findings highlight coding variants within B cell-associated genes that may contribute to altered immune signaling and underpin SLE pathogenesis in AFR women.

## Subjects and methods

### Data analysis

RNA-seq data, including cohort composition and detailed cell sorting data, were previously described^9^ and are available from the NCBI GEO database (https://www.ncbi.nlm.nih.gov/geo/) under accession GSE118254. Fastq files for each cell type were mapped to the hg38 reference genome using STAR v2.7.0.^32^ Duplicate reads were marked using PICARD MarkDuplicates v2.23.8 (Broad Institute) and removed from downstream analysis. The resulting BAM files were merged for all cell types for each donor into a single BAM file using samtools v1.11.^33^ Variants were identified from the merged BAM files using samtools v1.11 and bcftools v1.8.^33^ Variant calls were selected based on Phred quality score greater than 20 (QUAL > 20) and read depth less than 100 (DP < 100). GATK Funcotator v1.9.0^34^ was used to annotate variants with the hg38 gene level and variant effects annotations.^3^

### Clinical enrichment analysis

Mutation Annotation Format (MAF) files generated for individual donors were merged into a single combined MAF file to enable cohort-level analysis. Clinical enrichment analysis was performed on the merged MAF file using the clinicalEnrichment function of the maftools v2.18.0^35^ in R to identify genes with enriched mutations within the groupwise comparison—SLE vs HC. Genes with *P* value < 0.1 were selected for downstream analysis and visualization.

### Ancestry annotation

Minor allele frequencies for the 526 variants with dbSNP annotations were computed from the dbSNP build 151 released in 2021.^36^ K-means clustering was applied to the minor allele frequency data set using the biganalytics R package.^37^

### Expression analysis

Genes differentially expressed between SLE and HC were defined previously.^9^ DEG and variant-containing genes were overlapped based on ENTREZ ID and data visualized in R.

## Results

### Identification of single nucleotide polymorphisms in B cell subsets in an SLE cohort

**Figure 1:**
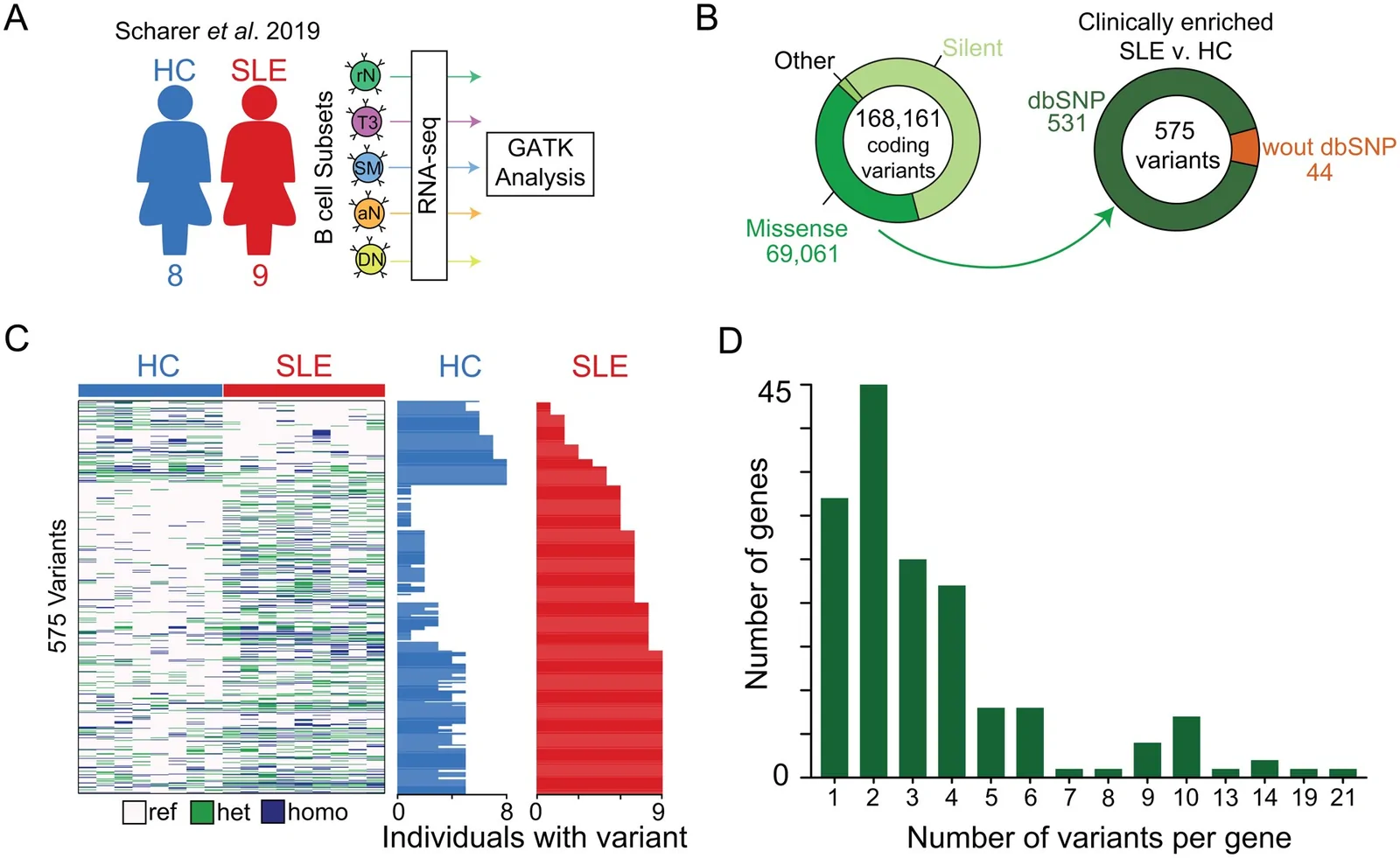
Identification of 575 missense clinically enriched coding single nucleotide polymorphisms in AFR SLE cohort. A. Schematic of the experimental design. Peripheral blood mononuclear cells (PBMCs) were obtained from healthy (HC, *n* = 8) and SLE (*n* = 9) individuals, as previously described.^9^ B cell subsets were isolated and subjected to RNA-seq followed by a variant-calling and analysis pipeline. B. Distribution of identified variants classified by functional annotation in all individuals (left) or the 575 clinically enriched missense variants (right). Clinically enriched variants (right) are colored based on prior annotation (green) or absence (orange) in dbSNP.^36^ C. Heatmap depicting the genotype of the 575 missense variants in which columns represent individuals grouped into either HC (blue) or SLE (red). Each row represents a variant, and genotype is color coded to reference (white, ref), heterozygous (green, het), or homozygous (navy, homo). Bar plots (right) indicate the number of individuals in either HC or SLE cohorts who contain the non-reference variants D. Bar chart depicting the number of variants per gene for the 158 genes containing missense variants.

Given that SLE disproportionately affects women of AFR ancestry with more severity and B cell-associated genetic variants are implicated in disease susceptibility, we aimed to further explore the B cell compartment of AFR females with SLE to identify polymorphisms with potential functional consequences. Here, we utilized transcriptome data from a previously published RNA-seq analysis comprising AFR women with SLE (*n* = 9) and sex- and ancestry-matched AFR healthy control donors (*n* = 8).^9^ This analysis spanned 5 circulating primary human B cell subsets: resting naive (rN; CD19^+^IgD^+^CD27^–^MTG^–^CD24^+^CD38^+^), transitional 3 (T3; CD19^+^IgD^+^CD27^−^MTG^+^CD24^mid/+^CD38^−^), activated naive (aN; CD19^+^IgD^+^CD27^−^MTG^+^CD24^−^CD38^−^), switched memory (SM; CD19^+^IgD^−^CD27^+^), and double negative (DN2; CD19^+^IgD^−^CD27^−^CXCR5^−^). These cell types encompass distinct stages of differentiation as cells mature through varying states of activation and differentiation to PC.^38^ The data were first merged across subsets, and variant calling was performed using the Genome Analysis Toolkit (GATK) RNA-seq variant discovery pipeline^34^ (Fig. 1A).

GATK analysis identified a total of 4,783,903 genetic variants across all patients at homozygous and heterozygous allele frequencies. Variants were annotated for predicted functional consequences using the GATK Funcotator tool. A majority of variants occurred in intronic regions, with only 168,161 variants (3.52% of total) present in the coding regions (Fig. 1B). Of the coding variants identified, 95,962 (57%) were silent, and 69,061 (41%) were missense variants. The remaining ∼2% represented nonsense, nonstop, start codon variants (start codon insertion and start codon SNP), frameshift variants (frameshift insertions and deletions), in-frame variants (in-frame insertions and deletions), and de novo start variants (start in-frame and out-of-frame) (Table 1). To identify genes harboring differential variant frequencies, a clinical enrichment analysis was performed using Maftools^35^ to compare the distribution of missense variants between SLE and HC cohorts. This analysis identified 170 genes exhibiting significant differences in variant frequencies between SLE and HC (*P *< 0.1). Among these, 158 genes contained a total of 575 distinct missense variants, while the remaining 12 genes harbored variants of other classes, including nonsense, frameshift, and indel. Of the 575 missense variants, 531 (92.3%) variants are present in the dbSNP database^36^ at the time of query (Fig. 1B). These results provide a distinct set of polymorphisms that may contribute to disease-associated B cell functions.

**Table 1:** Functional classification of 168,161 coding variants identified.

Variant classification	Number of variants
**Silent**	95,962
**Missense**	69,061
**Nonsense**	336
**Nonstop**	106
**Start Codon Insertion**	5
**Start Codon SNP**	71
**Frameshift Deletion**	331
**Frameshift Insertion**	600
**In-Frame Deletion**	398
**In-Frame Insertion**	290
**De novo (start in frame)**	379
**De novo (start out frame)**	622

To gain deeper insights into the distribution of the 575 missense coding variants identified between the SLE and HC cohort, variant genotypes were visualized as a matrix representing reference, heterozygous, and homozygous alleles for each individual (Fig. 1C). Overall, variants were observed across both SLE and HC cohorts, with most occurring as heterozygous alleles. Notably, 35% of variants were detected across all 9 AFR SLE individuals, indicating a large subset were shared within the SLE cohort. Furthermore, 6% were enriched exclusively in the AFR SLE cohort and were not present in HC, whereas only 2 variants from a single gene (*CXCL16*) were observed only in HC individuals. Additionally, variant distribution across the 158 genes containing missense polymorphisms revealed that 80% harbored two or more variants (Fig. 1D), suggesting that there may be multiple coding disruptions occurring within an individual gene that potentially lead to functional consequences in B cells. Together, these findings define a subset of coding variants enriched in SLE individuals and concentrated within specific genes that may underpin and drive alterations to B cell function within disease.

### SLE variants are enriched across African ancestral populations

**Figure 2:**
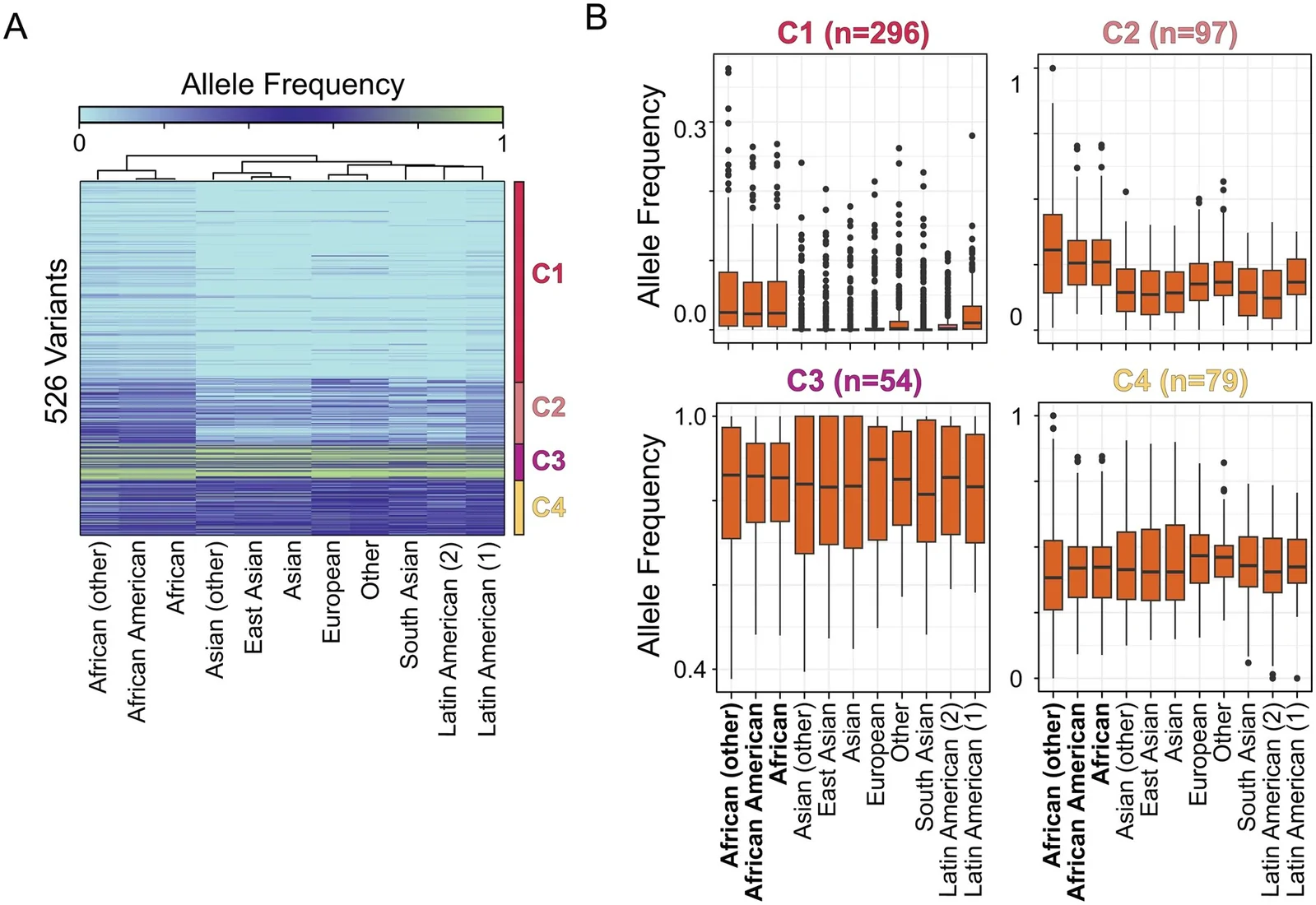
SLE variants are prevalent in African ancestry populations. A. Heatmap showing the allelic frequency of 526 missense variants across different ancestral populations. Each row represents a variant, and each column an ancestral group. Allele frequencies are grouped by *k*-means clustering (C1, C2, C3, or C4). B. Distribution of allele frequencies within each *k*-cluster. The center line (black) indicates the median value for each ancestral population. Boxes represent the interquartile range (orange), and individual data points are shown, with outliers plotted. African ancestry groups are bolded.

To determine whether the variants identified in our AFR SLE cohort exhibit ancestry-specific frequency patterns, allele frequencies were assessed across global populations using the dbSNP database.^36^ Of the 575 missense variants identified in our cohort, 526 were annotated in dbSNP and contained allele frequency (AF) data across multiple ancestral populations in the NCBI ALFA Allele Frequency database^36^ (Fig. 2A). AF were evaluated across major ancestry groups including African, Asian, European, Latin American, and other populations and subsequently clustered into 4 distinct polymorphism patterns describing the AF distribution across populations (C1–C4). Cluster 1 (C1) contained the largest number of variants, which exhibited relatively low AF across all populations (Fig. 2B). However, all three African ancestry groups contained higher AF than other populations, suggesting these SLE associated alleles were specific for AFR individuals. C2 displayed a similar pattern to C1, with low but higher overall AF and AFR ancestry containing the highest AF compared to others. In contrast, cluster 3 (C3) and 4 (C4) contained the smallest number of variants, but displayed the highest AF across ancestral populations, indicating these are more common variants. Together, these data indicate that the majority of variants identified in this study are enriched in AFR and may contribute to SLE uniquely in AFR populations compared to other ancestry groups.

#### Variant-associated genes exhibit altered transcriptional patterns in AFR SLE B cells

**Figure 3:**
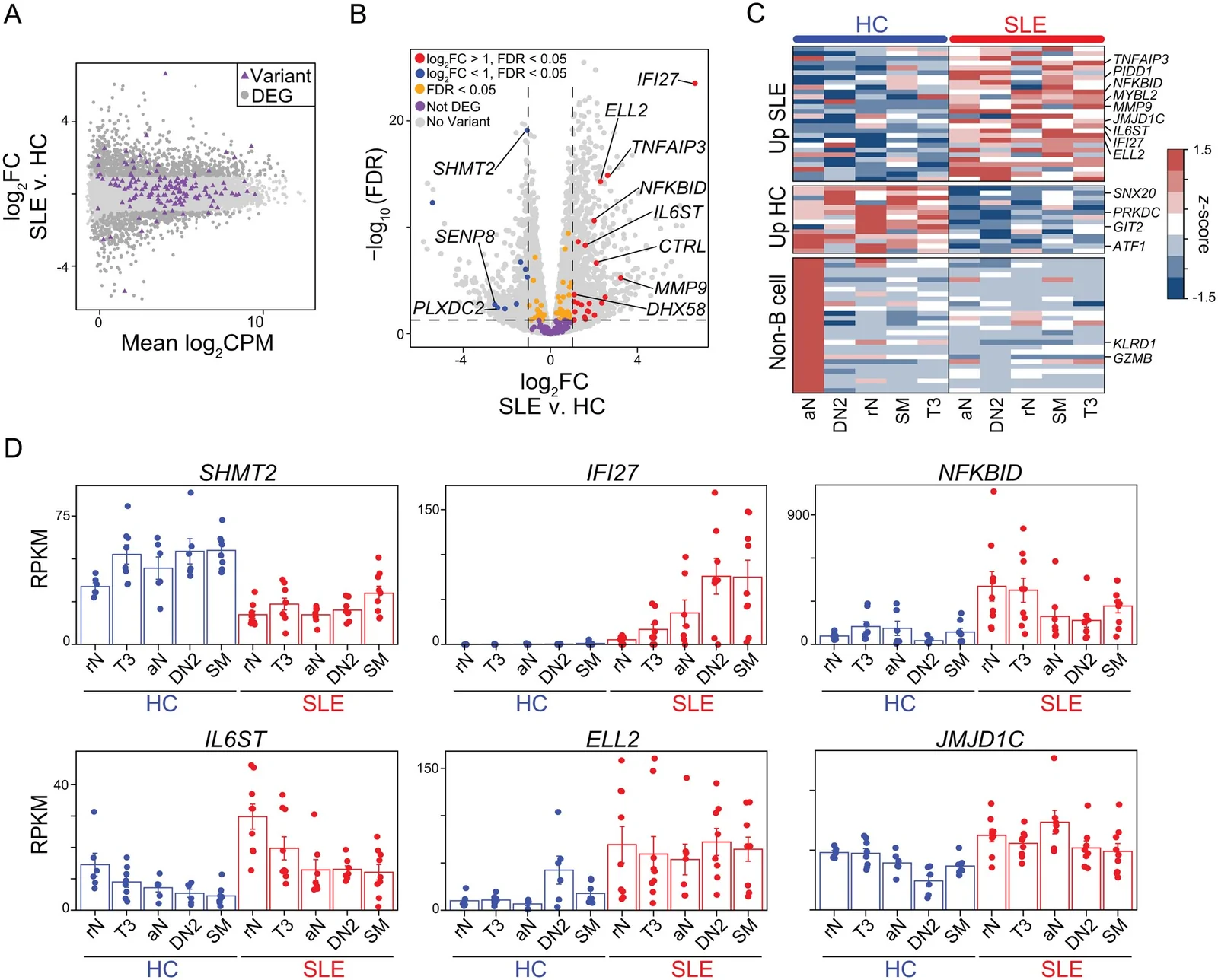
Variant-associated gene expression in SLE and HC B cell subsets. A. MA plot comparing mean log_2_ counts per million (CPM) and log_2_FC (SLE vs. HC) gene expression differences across all B cell subsets.^9^ Dark gray dots represent DEG between SLE and HC, whereas light grey dots denote non-DEG. Purple triangles represent variant containing genes. B. Volcano plot showing DEG between AFR SLE and HC B cells. Dashed horizontal line show significance thresholds for DEG with vertical lines denoting genes exceeding an absolute log_2_FC threshold ≥ 1. Genes containing variants that are not DEG are colored purple. C. Heatmap showing expression of variant-containing genes across all five B cell subsets (rN, T3, aN, DN2, and SM) in HC and AFR SLE individuals. Genes are grouped by enrichment in SLE, HC, and non-B cell lineage genes. Expression values are *z*-scored normalized and select genes are labeled. D. Bar plots showing the normalized expression data of examples of genes containing variants. RPKM, reads per kilobase per million transcripts. Error bars represent ± SEM.

To explore the potential functional consequences of the detected variants, we next examined the 158 missense variant harboring genes to determine whether they exhibited altered transcriptional patterns in AFR SLE B cells compared to HC. Previous analysis of the RNA-seq data integrating the 5 sorted B cell subsets from AFR SLE and HC defined 5,090 differentially expressed genes (DEG; FDR < 0.05), identifying DEG that were commonly changed across all cell types out of a total of 14,315 detected expressed genes^9^ (Fig. 3A). The majority of variant harboring genes did not meet the DEG threshold, indicating that variants enriched in AFR SLE B cells were not generally associated with significant transcriptional changes. Only 70 missense variant containing genes were DEG with large fold-changes between AFR SLE and HC (Fig. 3B). Several genes containing missense coding polymorphisms displayed large transcriptional changes including *IFI27*, *ELL2*, *TNFAIP3*, *NFKBID*, and *IL6ST*, and were more highly expressed in AFR SLE B cells. In contrast, a smaller subset of variant-containing genes, including *SHMT2*, had higher expression in HC B cells. These findings indicate that a subset of clinically enriched polymorphisms occur within genes that also show altered transcriptional activity in the AFR SLE B cell compartment.

Previous studies have associated SLE with altered B cell differentiation, including an expansion of aN and DN2 B cell subsets that contribute to the extrafollicular B cell activation and autoantibody production.^30^^,^^39–41^ To determine whether the transcriptional differences observed between AFR SLE and HC are driven by specific cell subsets or shared across the B cell compartment, we organized 70 variant-containing genes according to their enrichment in SLE or HC (Fig. 3C). Notably, the expression changes in these variant-containing genes, whether up- or downregulated in SLE, were shared across all five B cell subsets. In contrast, non-B cell associated genes, such as *KLRD1* and *GZMB*, showed inconsistent expression across B cell populations. Overall, this result suggests that variant-associated transcriptional effects occur broadly across the B cell compartment rather than within a specific subset. Additionally, representative genes delineate these shared patterns across B cell subsets (Fig. 3D). For example, *SHMT2*, associated with metabolism, showed higher expression in HC, whereas *IFI27*, an interferon-stimulated gene expression increased as B cells move toward terminal differentiation including DN2 and SM. Furthermore, *NFKBID*, a regulator of NF-κB signaling in B cells, was consistently upregulated in SLE across multiple subsets but was most highly expressed in more naive B cell subsets including rN and T3. Additional DEG that were increased in SLE compared to HC across all 5 B cell subsets and are important for B cell activation, differentiation, and function include *IL6ST*, *ELL2,* and *JMJD1C*. These data indicate that SLE DEG also contain disease associated AFR polymorphisms.

### TNFAIP3 variant rs2230926 is enriched in AFR SLE

**Figure 4:**
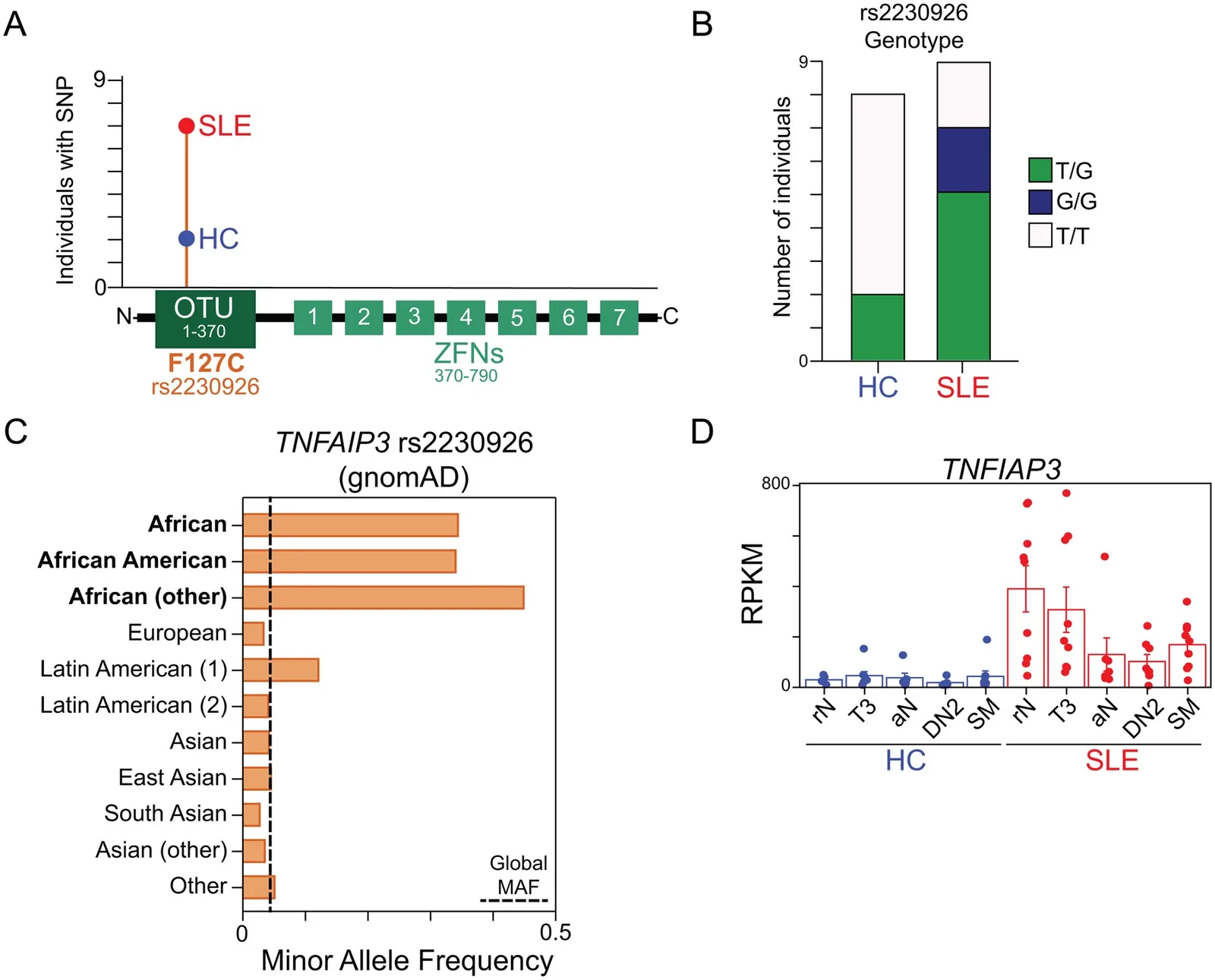
The *TNFAIP3* variant rs2230926 is enriched in AFR SLE B cells compared to HC. A. Schematic representation of *TNFAIP3* (A20) protein structure highlighting the location of the rs2230926 variant, which results in an F127C substitution within the N-terminus of the OTU domain (dark green). The location of zinc finger domains (ZFNs) are indicated in light green. The number of individuals carrying the variant allele within the AFR SLE (7 of 9) and HC (2 of 8) are indicated by red and blue dots, respectively. B. Genotype distribution analysis of rs2230926 in HC and AFR SLE cohorts. C. Minor allele frequency (MAF) of rs2230926 across global populations based on data from gnomAD.^46^ Bars represent population-specific allele frequencies for the indicated ancestry groups. The dashed line indicates the global MAF. D. Bar plot of *TNFAIP3* expression across B cell subsets from HC (blue) and AFR SLE (red) individuals. RPKM, reads per kilobase per million transcripts. Error bars represent ± SEM.

Among the variant-coding genes identified, *TNFAIP3*, encoding the A20 protein, emerged as a gene of particular interest due to its established role as a negative regulator of NF-κB signaling in B cells.^42–44^ The variant identified in our cohort, rs2230926, encodes a F127C (c.380T > G) substitution within the N-terminal ovarian tumor (OTU) domain of A20,^45^ and was detected in 7 of 9 AFR SLE individuals and 2 of 8 HC individuals within our cohort (Fig. 4A). Consistent with this observation, genotype analysis of the AFR SLE cohort revealed 2 individuals with the reference genotype (T/T), 2 with the homozygous variant genotype (G/G), and 5 with the heterozygous genotype (T/G). In contrast, the HC cohort contained six individuals with the reference genotype and two individuals with the heterozygous genotype (Fig. 4B). Utilizing the previously published Systemic Lupus Erythematosus Disease Activity Index (SLEDAI) scores for this cohort,^9^ we analyzed if there was an association with the F127C variant and disease activity. The rs2230926 variant was present in the 7 individuals with a range of SLEDAI scores from 4 to 22 and the 2 without the variant had SLEDAI scores of 6 and 10, resulting in no statistically significant association with disease activity. To determine the distribution across ancestral populations, we assessed the AF of rs2230926 across global populations using data from gnomAD^46^ (Fig. 4C). The global minor allele frequency (MAF) for the G allele was 0.044, indicating it is generally a rare allele in the human population. Conversely, the MAF in AFR populations was substantially higher, with frequencies of 0.346 in African, 0.342 in African American, and 0.451 in African (other) populations, demonstrating a ∼8.6-fold increase compared with the global average. We next evaluated *TNFAIP3* expression across B cell subsets in HC and AFR SLE. *TNFAIP3* expression was markedly elevated from AFR SLE individuals compared with HC across the 5 B cell subsets (Fig. 4D). Overall, these findings identify rs2230926 as a coding *TNFAIP3* variant enriched in women of AFR with SLE and associated with elevated *TNFAIP3* expression across B cell subsets.

## Discussion

SLE is a complex autoimmune disease characterized by dysregulated immune responses and the production of autoantibodies, with B cells playing a central role in disease pathogenesis.^39^^,^^47^^,^^48^ Genetic studies have identified numerous susceptibility loci associated with SLE; however, many of these studies have focused on bulk genomic variation and have not specifically examined how coding variants can influence distinct immune cell compartments, including B cells.^14^^,^^49^ The importance of B cells to SLE is underscored by recent studies reporting high success rates depleting B cells using CAR-T therapy.^50^ Therefore, we hypothesized that the B cell transcriptome within a high disease activity cohort may harbor disease associated variants that directly impact B cell function and disease pathogenesis. In this study, RNA-seq data representing multiple circulating B cell subsets from AFR women with high disease activity SLE was analyzed and revealed a set of missense coding variants enriched in AFR SLE B cells, many of which occur within genes involved in immune signaling pathways.

Our analysis identified 575 coding variants across 158 unique genes and were further characterized for their distribution across different global ancestral populations. One of the intriguing observations from our analysis was that most of the variants identified were not located within genes differentially expressed between AFR SLE and HC. While our analysis prioritized variant-containing genes that were also more highly expressed in AFR SLE, variants not categorized as DEG could still have important functional consequences and would not be prioritized in transcriptional studies. Additionally, the non-DEG missense variant containing genes could be DEG in other cell types or B cell contexts that were not analyzed here. In contrast, a subset of variant containing genes was upregulated in AFR SLE compared to HC, including *IFI27*, *NFKBID*, *IL6ST*, and *TNFAIP3.* Of interest, *IFI27*, a known biomarker of SLE and regulator of interferon (IFN) signaling was upregulated in AFR SLE.^51^^,^^52^ This enrichment was noted across all B cell subsets within the AFR SLE cohort and minimal expression in HC was consistent with the well-established type 1 IFN signature in SLE.^53^ Even though the enrichment was consistent across cell types, expression of *IFI27* was highly increased in more terminally differentiated B cells such as DN2 and SM compared to rN and T3.

Moreover, several genes, including *NFKBID* and *TNFAIP3,* are hallmark genes associated with the regulation of NF-κB signaling and differentially expressed in AFR SLE. In B cells, NF-κB regulates cellular processes such as survival, fate decisions, activation, and maintenance of cells and dysregulation of NF-κB signaling can contribute to immune responses.^54–56^ Specifically, *TNFAIP3* encoding the A20 protein, functions to suppress NF-κB activity through ubiquitin-editing mechanisms that terminate inflammatory signaling downstream of B cell immune receptor activation.^57^ The loss of A20 function, clinically associated with A20 haploinsufficiency, results in uncontrolled NF-κB signaling and heightened immune responses that can contribute to autoimmune diseases.^58–60^ In our data set, the rs2230926 polymorphism, which results in a F127C (c.380T>G) substitution within the OTU domain of *TNFAIP3*, was enriched in AFR SLE compared to SLE. Multiple GWAS studies have associated rs2230926 with autoimmune diseases such as rheumatoid arthritis and Sjögren syndrome,^42^^,^^43^ including one in AFR populations.^61^ Studies identifying functional consequences of rs2230926 report the inability of *TNFAIP3* to disassemble the K63 polyubiquitin chains that are required to inhibit canonical NF-κB signaling leading to excess inflammation;^45^^,^^62–64^ however, the impact on NF-κB signaling is mild compared to other A20 mutations.^65^ Importantly, these studies were conducted in HEK293T or epithelial cells, which may not accurately reflect the role of the F127C substitution in B cells. Therefore, within B cells, such alterations in A20 function may lower signaling thresholds or prolong NF-κB activation, potentially promoting aberrant B cell activation, the production of autoantibodies, and the skewing of B cell subsets contributing to disease pathology in SLE.

SLE disproportionately affects Black women, who experience 2–3 times higher incidence rates compared with White women.^6^^,^^66^ In addition to higher prevalence, AFR individuals often present with an increased risk of clinical manifestations and earlier onset of disease.^21^^,^^66^ Despite this increased disease burden, individuals of African ancestry remain underrepresented in genetic studies and relatively few ancestry-specific risk loci have been identified compared with other populations.^12^^,^^20^^,^^67^ This has limited our understanding of how population-specific genetic variation can predispose AFR individuals to SLE. Consistent with this, analysis of population allele frequencies revealed that the majority of coding variants, specifically in clusters 1 and 2 identified in our cohort, occur at higher frequencies in AFR compared to other global populations. These findings suggest that a subset of variants present in AFR SLE B cells may reflect ancestry-enriched polymorphisms that could contribute to disease susceptibility and immune dysregulation.

There are limitations that should be considered when interpreting these findings. First, this study focuses on AFR SLE women of varying levels of disease activity with SLEDAI scores of 4–22.^9^ Second, variability in treatment regimens among the cohort at the time of sample collection may represent a confounding factor, which can directly influence B cell activation states and transcriptional profiles.^68^ Third, the size of this cohort may limit statistical power to detect all significantly enriched SLE associated variants. Therefore, we chose to focus on variants previously described to have SLE associations but were new to AFR cohorts. It will be important going forward to expand these studies to profile larger cohorts that may also allow identification of coding and non-coding AFR SLE associations.

In summary, this study defines variant-associated genes within the B cell compartment of an underrepresented ancestral population who at the highest risk for SLE. These findings highlight the importance of examining genetic variation within B cells to identify polymorphisms that may affect the function of cellular activation, immune signaling, and immune responses. Together, this work provides insight into how coding variation within the B cell compartment may underpin B cell dysfunction that is a hallmark of disease pathogenesis for individuals most likely to develop SLE.

## Data Availability

RNA-seq data analyzed in this study are available from the NCBI GEO database (https://www.ncbi.nlm.nih.gov/geo/) under accession GSE118254.

## Abbreviations

AF: Allele frequency
AFR: African ancestry
aN: Activated naive
DEG: Differentially expressed genes
DN2: Double negative 2
EF: Extrafollicular
GATK: Genome analysis Toolkit
GWAS: Genome wide associations studie
HC: Healthy control
PC: Plasma cell
rN: Resting naive
SLE: Systemic Lupus Erythematosus
SLEDAI: Systemic Lupus Erythematosus Disease Activity Index
SM: Switched memory
T3: Transitional 3
